# RhoGAP RGA-8 supports morphogenesis in *C. elegans* by polarizing epithelia

**DOI:** 10.1242/bio.056911

**Published:** 2020-11-26

**Authors:** Hamidah Raduwan, Shashikala Sasidharan, Luigy Cordova Burgos, Andre G. Wallace, Martha C. Soto

**Affiliations:** 1 Department of Pathology and Laboratory Medicine, Rutgers – RWJMS, Piscataway, NJ 08854, USA; 2 Cell and Developmental Biology Graduate Program, School of Graduate Studies, Rutgers – RWJMS, Piscataway, NJ 08854, USA; 3 Department of Biological Sciences, Fairleigh Dickinson University, Teaneck, NJ 07666, USA

**Keywords:** Morphogenesis, RhoGAP, CDC-42, Cell migration

## Abstract

CDC-42 regulation of non-muscle myosin/NMY-2 is required for polarity maintenance in the one-cell embryo of *Caenorhabditis elegans*. CDC-42 and NMY-2 regulate polarity throughout embryogenesis, but their contribution to later events of morphogenesis are less understood. We have shown that epidermal enclosure requires the GTPase CED-10/Rac1 and WAVE/Scar complex, its effector, to promote protrusions that drive enclosure through the branch actin regulator Arp2/3. Our analysis here of RGA-8, a homolog of SH3BP1/Rich1/ARHGAP17/Nadrin, with BAR and RhoGAP motifs, suggests it regulates CDC-42, so that actin and myosin/NMY-2 promote ventral enclosure during embryonic morphogenesis. Genetic and molecular data suggest RGA-8 regulates CDC-42, and phenocopies the CDC-42 pathway regulators WASP-1/WSP-1 and the F-BAR proteins TOCA-1 and TOCA-2. Live imaging shows RGA-8 and WSP-1 enrich myosin and regulate F-actin in migrating epidermal cells during ventral enclosure. Loss of RGA-8 alters membrane recruitment of active CDC-42. We propose TOCA proteins and RGA-8 use BAR domains to localize and regenerate CDC-42 activity, thus regulating F-actin levels, through the branched actin regulator WSP-1, and myosin enrichment. RhoGAP RGA-8 thus polarizes epithelia, to promote cell migrations and cell shape changes of embryonic morphogenesis.

## Abstract (precis)

**Summary:** RGA-8, a protein with membrane binding and actin regulatory motifs, promotes embryonic morphogenesis by localizing active CDC-42 in developing epithelia, and controlling actin and actin motors during cell movements.

## INTRODUCTION

Organ and tissue formation are highly regulated processes during embryonic development. During embryogenesis, epithelial cells must develop and maintain apicobasal polarity and healthy cell–cell junctions as they move past or over other tissues in the process of morphogenesis. Defects in this process can lead to birth defects, or premature death. Epidermal morphogenesis in the nematode *Caenorhabditis elegans* is an ideal model to study tissue morphogenesis (Sulston et al., 1983; Priess and Hirsh, 1986; Chisholm and Hardin, 2005).

**Fig. 1.**
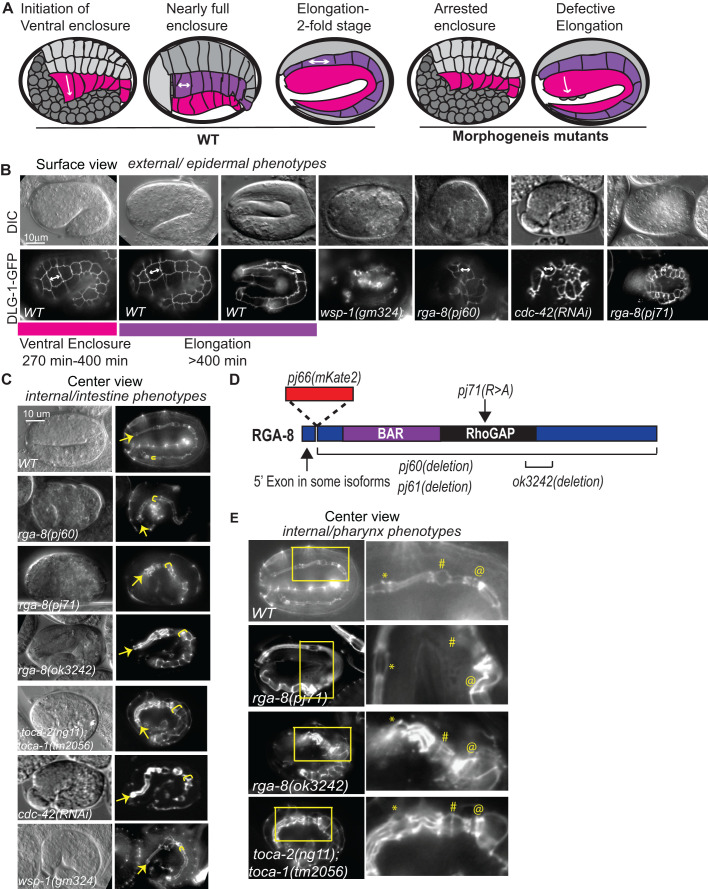
**RhoGAP RGA-8 and CDC-42 pathway genes regulate two stages of morphogenesis.** (A) Epidermal morphogenesis in *C. elegans* requires regulated movements of the cells of the ventral (magenta) and lateral (purple) rows. Ventral enclosure requires ventral-ward migrations (magenta) while during early elongation, cell shape changes in the lateral seam cells (purple) increase anterior-posterior length. Morphogenesis mutants arrest at either or both stages. (B) Wild-type embryos shown beginning at 270 min after first cleavage, by differential interference contrast (DIC, top), and *dlg-1::gfp* (bottom) (Totong et al., 2007), with a focus on the seam cells, illustrate cell shape changes of elongation. Mutant embryos are shown at the same time and focal plane. White arrow marks the same seam cell. (C) Wild-type and mutant embryos at 390 min after first cleavage, with a focus on the internal epithelia, illustrate widening and distortion of apical regions of the pharynx and intestine in the mutants. Yellow arrow: anterior pharynx; yellow bracket: anterior intestine. (D) RGA-8 molecular model illustrates conserved BAR and RhoGAP domains. Genetic mutations from the *C. elegans* Gene Knockout Consortium (*ok3242* deletion) or from our CRISPR studies are indicated: *pj60*, *pj61* deletions, *pj71* GAP mutant and *pj66* endogenous CRISPR N-terminal GFP tag, OX681 *rga-8*(*pj66*)[*mKate2::rga-8*] (see Figs 2, 3 and 4). (E) Comparison of the effects of *rga-8* mutations and *toca-2; toca-1* mutants on pharynx morphogenesis. In the enlarged region, * and # mark the anterior and posterior bulbs of the pharynx, while @ marks the valve cells at the junction between pharynx and intestine. In this figure and others embryos are shown anterior to the left, and dorsal up, unless otherwise stated. Scale bars in all figures are 10 μm long. Embryonic times represent minutes after first cleavage at 23°C. Embryos were cultured and imaged at 23°C, unless otherwise stated.

Epidermal morphogenesis in *C. elegans* can be divided into several stages. Epidermal cells are born at the posterior and dorsal surface of the embryo and become arranged in three types of adjacent cells with distinct behaviors: two rows of dorsal cells, and on each side of the embryo, two rows of lateral seam cells, and two rows of ventral cells (Fig. 1A). Epidermal morphogenesis begins when two outer rows, right and left ventral epidermal cells, migrate ventrally, pulling along other epidermal rows to enclose the embryo, in a process known as ventral enclosure. This tissue migration is led by the two anterior-most ventral cells on each side, the leading cells. The leading cells reach the ventral midline first, while the more posterior ventral cells, the pocket cells, undergo a purse-like constriction, to enclose the embryo on the ventral side. Simultaneously, the two dorsal rows undergo dorsal intercalation to generate a single row of cells, in a process analogous to vertebrate convergent extension. Epidermal elongation, the process that squeezes the embryo into a worm shape, begins when the two rows of lateral seam cells increase their length along the anterior to posterior axis, and decrease along the dorsal/ventral axis (Fig. 1A). The dynamics of actin and actomyosin contractility that regulate epidermal morphogenesis are regulated by the Rho GTPases.

In *C. elegans* three major Rho GTPases, Rac1/CED-10, CDC-42 and RhoA/RHO-1, have been shown to be involved in some aspects of epidermal morphogenesis. Ventral enclosure is regulated by the GTPase Rac1/CED-10, which activates WAVE/Scar, a nucleation promoting factor (NPF) that activates branched actin formation through Arp2/3 (Soto et al., 2002; Sawa et al., 2003; Patel et al., 2008). Rac1/CED-10 and WAVE/Scar/WVE-1 also regulate dorsal intercalation (Soto et al., 2002; Patel et al., 2008; Walck-Shannon et al., 2015), underscoring the important role played by CED-10/Rac1 in regulating the overall process of epidermal morphogenesis. In parallel to the CED-10/Rac1/WAVE pathway, morphogenesis is supported by the CDC-42 GTPase and its effector, the NPF WASP/WSP-1 (Sawa et al., 2003; Withee et al., 2004; Walck-Shannon et al., 2016).

Epidermal elongation depends on regulated actomyosin driven by the GTPase RhoA/RHO-1 (Reviewed in Wissmann et al., 1999; Vuong-Brender et al., 2016). Actomyosin is negatively regulated by myosin phosphatase MYPT/MEL-11, which is expressed in the dorsal and ventral epidermis (Wissmann et al., 1999). In the absence of MEL-11 embryos burst due to increased tension on adherens junctions. Actomyosin contractility in the seam cells is promoted by Rho Kinase ROCK/LET-502, which is expressed in the epidermal seam cells (Wissmann et al., 1999). Removing ROCK/LET-502 caused defects in elongation of the embryo. Activation of myosin II is achieved mainly through ROCK/LET-502, but two additional kinases can contribute to maintain myosin II activity. P21-activated kinase PAK-1 and CDC-42-activated kinase MRCK-1 act in parallel to LET-502, since their loss enhances *let-502* mutant severity. MRCK-1 acts upstream of MEL-11 (Gally et al., 2009) and is under the regulation of CDC-42 (Gally et al., 2009).

Studies of *cdc-42* function in *C. elegans* have mostly focused on its role during the one-cell stage, where it helps set up anterior/posterior polarity as a component of the anterior PAR complex (Reviewed in Cowan and Hyman (2007). CDC-42 in the one-cell embryo also regulates myosin levels and cortical enrichment (Kumfer et al., 2010; Motegi and Sugimoto, 2006; Schonegg et al., 2007; Schonegg and Hyman, 2006; Beatty et al., 2013). Studying the morphogenesis role requires getting past the early roles for CDC-42 in setting up polarity. A role for CDC-42 and WASP/WSP-1 during epidermal morphogenesis has been proposed (Beatty et al., 2013; Sawa et al., 2003; Withee et al., 2004). Ouellette and colleagues found that overexpression of CDC-42 caused altered protrusions of leading cells, and embryonic lethality, suggesting a balance of active and inactive CDC-42 is required for efficient ventral migration (Ouellette et al., 2016). Transducer of cytokinesis-1, TOCA-1, contains a GTPase binding domain (GBD) that binds to CDC-42, an SH3 domain that binds to proline-rich region of WASP or other proteins, and an F-BAR domain that senses and binds to curved membranes (Takano et al., 2008) (Fig. 6B). *C. elegans* has two partially redundant homologs of TOCA1, named TOCA-1 and TOCA-2, which are both implicated in epidermal morphogenesis of *C. elegans* (Giuliani et al., 2009). The Rho GAP RGA-7 has been shown to genetically interact with TOCA-1/2 and CDC-42 during the formation of actin-rich protrusion in leading cells (Ouellette et al., 2016). These results suggested that the conserved pathway of branched actin regulation by TOCA-1/2- CDC-42-WASP also regulates ventral enclosure during *C. elegans* morphogenesis. Zilberman and colleagues used ZIF1 degron technology to partially rescue *cdc-42* null mutants to survive past the one-cell defects to study epidermal morphogenesis, and uncovered ventral enclosure and elongation defects in *cdc-42* mutants (2017). CDC-42 also supports dorsal intercalation (Walck-Shannon et al., 2016). These reports support a role for CDC-42 during epidermal morphogenesis.

The role actomyosin contractility plays during ventral migration is not clear. Anillin actin binding protein ANLN/ANI-1, a multidomain protein that organizes actomyosin contractility, is required to ensure proper alignment of contralateral leading-edge epidermal cells meeting at the ventral midline. However, expression of ANI-1 was not detected in the epidermal cells, leading to the suggestion that ANI-1's action may be due to the interaction between the underlying neuroblast (neuron precursors) with the migrating epidermal cells. Interestingly, loss of *rho-1, let-502* or *mel-11* led to ventral enclosure defects, but it was not clear if this was due to roles in the neuroblasts or in the epidermis (Fotopoulos et al., 2013). Careful imaging of non-muscle myosin, NMY-2::GFP, supported a role in the epidermis during ventral enclosure (Wernike et al., 2016). Studies of HUM-7, a GAP that regulates epidermal morphogenesis through RHO-1/RhoA, showed it affected NMY-2::GFP levels specifically in the migrating epidermal cells (Wallace et al., 2018). Altogether, the evidence suggests RHO-1/RhoA and myosin also contribute to ventral enclosure.

Here we investigate the morphogenesis role of the RhoGAP and BAR domain protein, RGA-8, the single *C. elegans* homolog of SH3BP1/Rich1/Nadrin/ARHGAP17, which are thought to act as GAPs for the GTPases Rac1 or CDC-42 (Cicchetti et al., 1995; Parrini et al., 2011; Huang et al., 2013; Beck et al., 2013; Chan Wah Hak et al., 2018). In the first characterization in *C. elegans* of this protein, we generate mutations in RGA-8 using CRISPR and compare the embryonic morphogenesis phenotypes to loss of function mutations in CDC-42 pathway genes including *wsp-1* and the *toca-2*(*ng11*)*;toca-1*(*tm2056*) double mutant, and then test genetic interactions. CRISPR-generated *rga-8* putative null alleles and GAP mutants are tested for effects on a CDC-42 biosensor. Using RGA-8 endogenously tagged via CRISPR, we examine RGA-8 distribution in polarized epithelia of the pharynx and intestine. *rga-8* mutants, and WASP/WSP-1 mutant *wsp-1(gm324)* are examined for effects on F-actin and non-muscle myosin II (NMY-2::GFP) at the leading edge of migrating epidermal cells during ventral enclosure. We propose a model where RGA-8, TOCA-1/TOCA-2, and WSP-1, working with CDC-42, enrich and maintain polarized NMY-2::GFP accumulation at the epidermal pocket cells and leading edge cells to support ventral enclosure.

## RESULTS

### RGA-8 is a GAP that regulates *C. elegans* embryonic morphogenesis

Our previous work investigated GTPases Rac and Rho regulation of epidermal morphogenesis (Soto et al., 2002; Wallace et al., 2018) (Fig. 1A). To identify additional regulators of GTPases during epidermal morphogenesis, we screened the *C. elegans* family of GTPase activating proteins (GAPs), which catalyze GTP hydrolysis, thus inactivating Rho GTPases (Neukomm et al., 2011). Loss of RhoGAP *rga-8* via RNAi led to embryonic lethality with defective morphogenesis (5% lethality, Table 1). Since the homologs of RGA-8, including SH3BP1, RICH-1 and NADRIN, are proposed GAPs for the GTPase Rac1, we further examined the effects of *rga-8* on embryonic morphogenesis using RNAi depletion, an existing *rga-8* mutation (*ok3242*) and new CRISPR alleles made for this study. We first examined the mutants for effect on morphogenesis (Table 1). All mutations in *rga-8* led to embryonic lethality due to morphogenesis defects, including arrest during ventral enclosure and elongation of epidermal morphogenesis, resulting in 4–13% of the embryos dying during embryogenesis, depending on the allele (Table 1). For example, *rga-8*(*pj60*) and (*pj61*), which contain a large deletion that removes all or most of *rga-8* (Fig. 1D), resulted in 3.4% and 4.5% embryonic lethality, with approximately half the embryos arresting during ventral enclosure, and the other half during elongation (Fig. 1, Table 1). *rga-8*(*pj71*)*,* a CRISPR allele which switches the catalytic Arginine to an Alanine, predicted to interfere with GAP function but preserve protein expression, resulted in higher lethality (13%). To better examine the phenotypes, all alleles were crossed into the Discs Large homolog, *dlg-1::gfp* transgenic strain (FT250; Totong et al., 2007), which is expressed at *C. elegans* apical junctions, to monitor changes in epithelial movements. We detected similar levels of embryonic lethality, and similar distribution of arrests at ventral enclosure and during elongation (Table 1). *rga-8*(*ok3242*), a 611 bp deletion causing a frame shift and premature stop, part way through the GAP domain (Fig. 1D), and *pj71*, a CRISPR GAP mutation, resulted in 8% and 11% embryonic lethality, respectively (Table 1), with defective ventral enclosure and elongation (Fig. 1B), and widening of the intestinal lumen (Fig. 1C) and pharyngeal lumen (Fig. 1E). Thus, loss of *rga-8* leads to lethality due to defects at two steps of epidermal morphogenesis, accompanied by widening of the apical lumen of two internal epithelia, and its RhoGAP domain appears to be involved in this process. While enclosure defects may contribute to elongation defects or internal epithelial defects, embryos that enclose and fail elongation also show the internal apical epithelial defects (Fig. 1B,C).

**Table 1.**
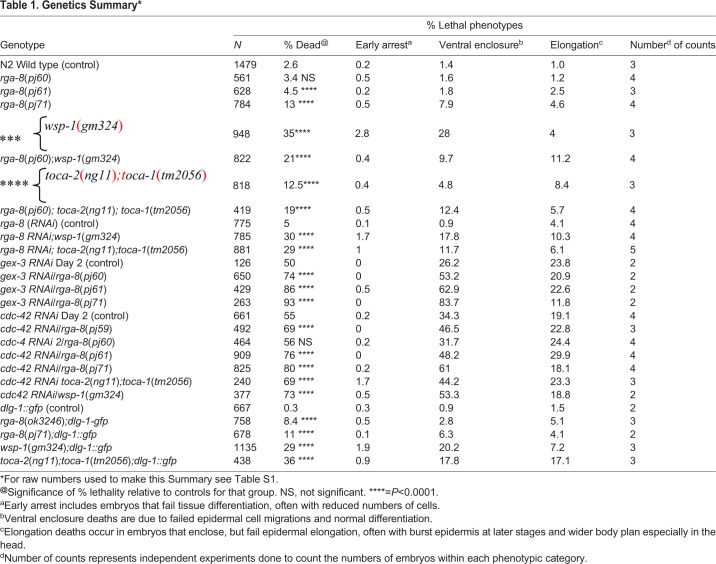
Genetics Summary*

We next investigated which GTPase may be regulated by RGA-8. If RGA-8 was a GAP for the GTPase Rac1/CED-10, we expected loss of *rga-8* to rescue the hypomorphic alleles of *ced-10* as has been shown, for example, for the RhoGAP SRGP-1 (Neukomm et al., 2011). Combining the *pj60, pj61 or pj71* mutants with partial depletion of the Rac-1-dependent WAVE complex, via *gex-3 RNAi*, (Patel et al., 2008), led to enhanced embryonic lethality, from 50% to 77–93%, depending on the allele (Table 1). Genetic doubles of *rga-8*(*pj60*) with the hypomorphic allele, *ced-10*(*n1993*) resulted in increased lethality from 11% to 16% (*n*=643, Table S1). This suggested that RGA-8 does not behave like a Rac1 GAP.

We tested RGA-8 genetic interactions with the RHO-1 pathway. Embryonic lethality due to RNAi of the Rho-1 kinase *let-502* was increased by the putative null allele *rga-8*(*pj60*) from 40% to 64%, and also increased by the *pj71* GAP mutant (Table S1). These results suggest *rga-8* acts in parallel to the RHO-1 pathway.

We next investigated if *rga-8* interacted with known components of *cdc-42* pathway: *toca-1, toca-2* and *wasp/wsp-1*. WASP/WSP-1 is a branched actin nucleation promoting factor that is auto-inhibited until it binds TOCA and CDC-42 (Fig. 6B). Activated WASP-1 promotes branched actin formation through the Arp-2/3 complex (Reviewed in Kurisu and Takenawa, 2009; Takenawa and Suetsugu, 2007). TOCAs are F-BAR domain proteins that recruit regulators of branched actin to subcellular domains (Fig. 6B). Deletion mutations *wsp-1*(*gm324*) and *toca-2*(*ng11*)*;toca-1*(*tm2056*) led to embryonic lethality during ventral enclosure and elongation at 35% and 13% respectively, as previously reported (Table 1, Fig. 1, Withee et al., 2004; Giuliani et al., 2009). When the *rga-8*(*pj60*) deletion was crossed to the *toca-2(ng11);toca-1(tm2056)* double mutant, it enhanced embryonic lethality additively from 13% to 19% (Table 1). In contrast, genetic doubles with *wsp-1*(*gm324*)*,* a known effector of *cdc-42* that regulates ventral enclosure (Sawa et al., 2003), resulted in intermediate embryonic lethality, closer to *wsp-1* levels, at approximately 21%*.* Similar results were seen when *rga-8* was depleted via RNAi in animals with *toca-2*(*ng11*)*;toca-1*(*tm2056*) (additive enhancement) and *wsp-1*(*gm324*) (similar to *wsp-1*) mutations (Table 1). These results suggested that RGA-8 may work in the CDC-42 pathway to regulate embryogenesis, possibly parallel to TOCA-1/TOCA-2, and with WSP-1.

### RGA-8 is expressed in most cells with enrichment at apical surfaces of pharyngeal and intestinal cells

**Fig. 2.**
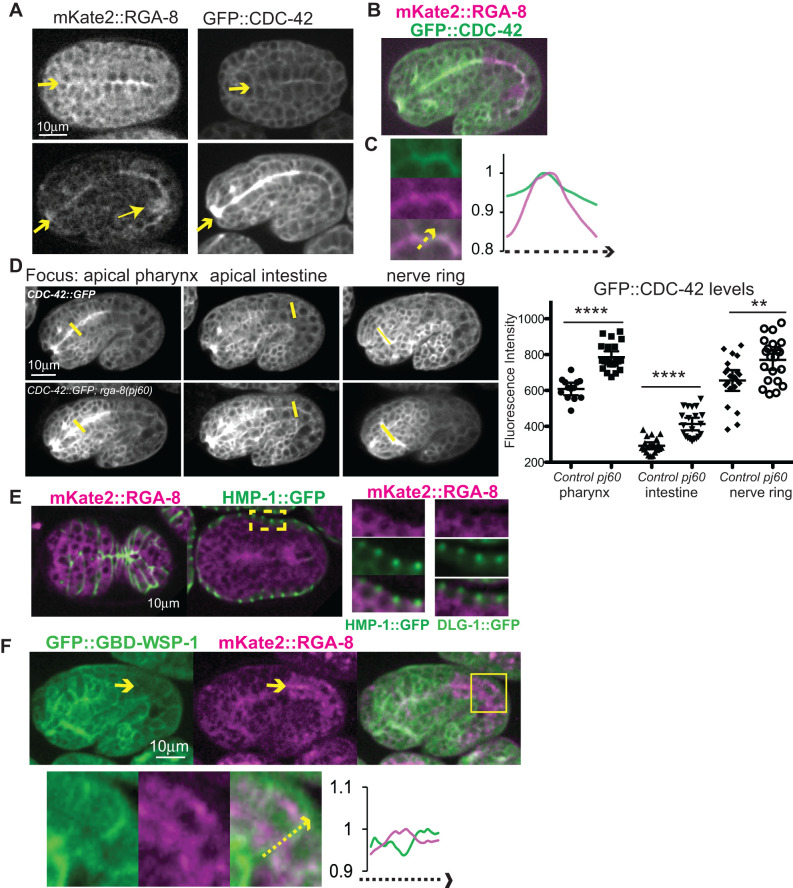
**RGA-8 is enriched apically in pharyngeal and intestinal epithelia.** (A–C) Embryos expressing *mKate2::rga-8*, or *gfp::cdc-42*, or both (color images). Embryos at 240 min, top row (A), and 360 min, bottom row (A and B). Arrows at anterior point to apical pharynx. Arrow head, lower left, indicates bright signal in the two germline cells. (C) a crop from the intestine in B, includes a line scan to compare enrichment at the apical intestine. (D) Control *gfp::cdc-42* embryos were compared to *gfp::cdc-42; rga-8*(*pj60*) embryos for enrichment at apical pharynx, intestine and at the nerve ring. Different focal planes are shown to better illustrate each tissue. (E) 300 min embryo shown ventral up, surface focus (left) or internal focus (right) as epidermis meets on the ventral side, expressing *mKate2::rga-8* and *hmp-1::gfp* (Marston et al., 2016) or *dlg-1::gfp* (Totong et al., 2007) to illustrate epidermal apical epithelial junctions. Yellow square indicates region cropped to focus on epidermis. (F) Embryos at 360 min, anterior left and dorsal up, focused on internal epithelia show localization of *mKate2::rga-8* relative to *gbd-wsp-1::gfp*. In crop of intestine a line scan (yellow arrow) compares apical enrichment. Statistical significance in D was determined by two-tailed Student's *t*-test with Welch's correction. **P<0.001, ****P<0.0001.

To examine the expression pattern of RGA-8, we endogenously tagged it at the N-terminus with mKate2, a red fluorophore, using CRISPR technology (Dickinson et al., 2015) (Fig. 1D). A caveat for interpreting this expression is that we were unable to confirm that the tagged CRISPR allele rescues lethality, since the beginning lethality is so low. The endogenously tagged allele was healthy (<1% lethality; Table S1). Confocal spinning disk microscopy showed diffuse signal in most cells, with enrichment of *mKate2::rga-8* at apical regions of two epithelia, pharynx and intestine (Fig. 2A,B). Apical enrichment starts at approximately 240 min after first cleavage, at a time when apical junctions are being established, and is maintained until adulthood. Apical enrichment in epithelia is also seen in the transgenic strain, *gfp::cdc-42* (Fig. 2A,B; Kumfer et al., 2010).

#### RGA-8 regulates apical enrichment of GFP::CDC-42

If RGA-8 is a GAP that regulates CDC-42, it may alter the levels of CDC-42. Crossing the *rga-8*(*pj60*) deletion allele into *gfp::cdc-42,* a transgene that rescues loss of *cdc-42* (WS4700; Neukomm et al., 2014), resulted in increased GFP::CDC-42 levels at the apical surfaces of the intestine and pharynx (Fig. 2D). Other regions with high GFP::CDC-42 expression, like the nerve ring, also showed higher expression in *rga-8*(*pj60*)*.* This result suggested that RGA-8 regulates CDC-42.

#### Epidermal expression

Since RGA-8 regulates ventral enclosure, we examined if it is expressed in the epidermis. *mKate2::rga-8* in epidermal cells appears to localize basally to two apical junction complexes as shown by comparison to the cadherin component alpha catenin/ HMP-1::GFP, and the DLG-1/AJM-1 (Apical Junction component 1 homolog) complex component, DLG-1::GFP (Fig. 2E). We conclude that RGA-8 is expressed in the epidermal cells, in a diffuse pattern that appears basal to the apical junctions.

### RGA-8 regulates membrane enrichment of a CDC-42 biosensor

If RGA-8 is a CDC-42 GAP, it may regulate the membrane distribution of active CDC-42. This was tested using a biosensor for active CDC-42, the *gfp-gbd-wsp-1* strain that contains the GBD of WSP-1, which can bind GTP-bound or active CDC-42 (Fig. 6B). This biosensor was verified with *in vivo* FRET/FLIM studies, and *in vitro* biochemical studies that showed it binds wild-type CDC-42, and constitutively active CDC-42(Q61L) but not constitutively inactive mutant CDC-42(T17N) (Kumfer et al., 2010). We first compared the expression pattern of *mKate2::rga-8* to *gfp-gbd-wsp-1*. In the pharynx, both transgenes were apically enriched, just like *gfp::cdc-42*. However, in the intestine, the two transgenes had a complementary pattern: while *mKate2::rga-8* was apically enriched (just like *gfp::cdc-42*), *gfp-gbd-wsp-1* was depleted from the apical region, and instead showed basolateral enrichment (Fig. 2F). This suggested active CDC-42 has polarized apical/basal distribution in two epithelia, the pharynx and intestine, but the pattern is distinct in each.

**Fig. 3.**
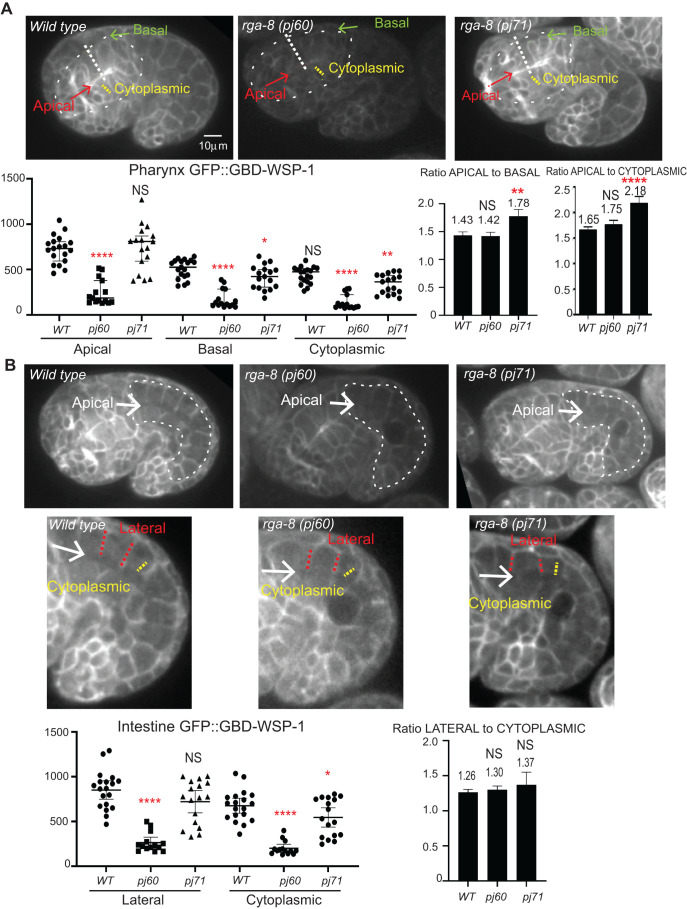
**RGA-8 regulates membrane enrichment of active CDC-42 in epithelia.** Distribution of *gbd-wsp-1::gfp* in control, *rga-8*(*pj60*) and *rga-8*(*pj71*) mutant embryos. (A) 360 min embryos, shown with focus on the apical pharynx (red arrow). Dotted outline of the pharynx is shown. (B) 360 min embryos, shown with focus on the apical intestine (while arrow); dotted white outlines the intestine. The intestinal area was enlarged and enhanced below the original images to better show the apical and basolateral pattern of *gbd-wsp-1::gfp* in the mutants. *n*=at least 14 embryos per type of measurements, and three measurements were averaged per embryo. Measured regions are indicated on each image using dotted lines. For A, apical and basal measurements resulted from three lines drawn per embryo, from apical to basal, and the average of the maximum intensity was plotted. For cytoplasmic measurements in A and B, the average of three average intensities was plotted. Lateral measurements in B are average intensities based on three measurements per embryo. Statistical significance for measurements was determined by one-way ANOVA with Tukey’s post-hoc test. The logarithm of the ratios was submitted to a two-tailed paired *t*-test between wild type and each mutant. *P<0.05, **P<0.001, ****P<0.00001.

To test if the apical/basal distribution, or membrane distribution, of active CDC-42 depends on RGA-8, we crossed the *rga-8* null allele *pj60* or the GAP point mutation *pj71,* into *gbd-wsp-1::gfp* and monitored the accumulation at the 1.5-fold stage (390 min after first cleavage) when the pharynx and intestine have acquired apical/basal polarity. In the pharynx, control embryos showed membrane enrichment of GBD-WSP-1::GFP, with higher enrichment at apical membranes relative to basal ones, so that the ratio of apical to basal levels was 1.43-fold, while the ratio of apical to cytoplasmic level was 1.65-fold. *rga-8*(*pj60)* embryos showed reduced overall signal, with reduced cytoplasmic, apical and basal membrane enrichment, while the ratio of apical to basal or of apical to cytoplasmic did not change significantly (1.42 and 1.75-fold, respectively). *rga-8*(*pj71*) GAP mutants showed a different result, with higher apical enrichment, and decreased basal and cytoplasmic levels, and significantly increased ratios of apical to basal or apical to cytoplasmic enrichment (1.78 and 2.18-fold, respectively) (Fig. 3A).

In the intestine, control embryos are basolaterally enriched for *gbd-wsp-1::gfp*, with a ratio of lateral to cytoplasmic signal of 1.26. Both *pj60* and *pj71* embryos show decreased *gbd-wsp-1::gfp* levels in the intestine, including decreased basolateral membrane signal and decreased cytoplasmic signal. The lateral to cytoplasmic ratio was not significantly changed for either allele (1.3 and 1.37-fold, respectively), while the drop in levels was significant (Fig. 3B). Thus, either complete loss, or a GAP mutation in RGA-8 resulted in altered membrane enrichment, and overall levels, of active CDC-42 in two polarized epithelial tissues, the pharynx and intestine. These tissues display apical morphogenesis defects when RGA-8 is missing, or has a mutated GAP domain (Fig. 1C,E). Thus, RGA-8 loss correlates with altered CDC-42 membrane enrichment, and altered apical morphogenesis.

To test if the CDC-42 biosensor could help us characterize the epidermal role of RGA-8 and CDC-42, we first examined the *gbd-wsp-1::gfp* pattern at the ventral epidermis during enclosure. *gbd-wsp-1::gfp* is enriched at cell boundaries, but we did not detect obvious enrichment at the ventral midline. We instead examined the distribution of F-actin and myosin in the ventral epidermis.

### Defects in CDC-42 pathway alter F-actin levels in migrating epidermis

**Fig. 4.**
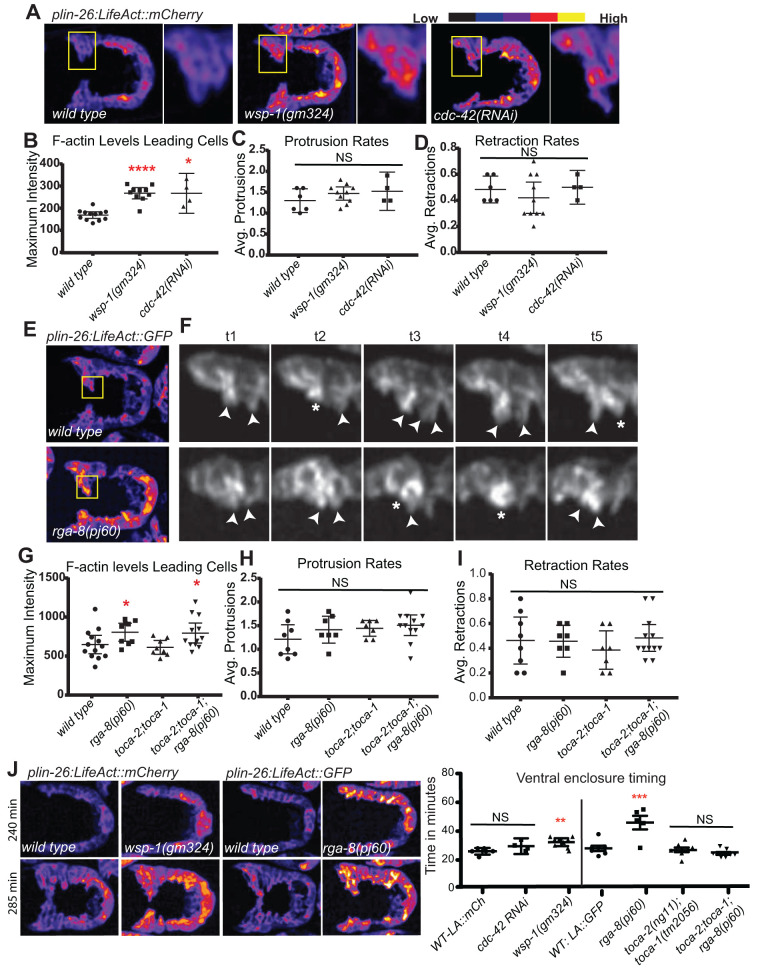
**RGA-8 and WSP-1 alter levels of epidermal F-actin in migrating cells.** (A,E) 270 min embryos shown ventral up, surface focus, were used to compare levels of *plin-26::LifeAct::mCherry* (A) or *plin-26::LifeAct::gfp* (E) (Gally et al., 2009; Havrylenko et al., 2015) in migrating leading-edge epidermal cells during ventral enclosure. Both strains were used since some mutants mapped closely to the insertion sites of these integrated transgenes. Embryos were imaged at 2 min intervals beginning at 240 min after first cleavage. Embryos were pseudocolored using the ‘Fire’ function in ImageJ, and intensity is shown from low (blue) to high (yellow). Yellow boxes enclose two leading cells, further amplified to the right. Levels (B,G): highest signal in the leading cells. Protrusions (C,H) and retractions (D,I) indicate average F-actin protrusions or retractions per time point in two leading cells during a 10 min period. (F) In two leading cells, arrowheads mark protrusions and asterisks mark retractions. (J) These same movies were used to measure the timing of ventral enclosure, from the beginning of ventral-ward protrusions until the cells met at the midline. Quantitation of *plin-26::LifeAct::gfp* or *plin-26::LifeAct::mCherry* plotted for mean with 95% confidence interval. ***P<0.001, ****P<0.0001.

The Rac/WAVE/Scar pathway is known to regulate F-actin levels, polarity and dynamics in migrating epidermal cells (Bernadskaya et al., 2012), but the effects of the CDC-42 pathway on F-actin in migrating epidermis is less understood. We monitored levels of epidermal F-actin by crossing genetic mutants into strains where LifeAct is under the LIN-26 promoter, expressed in all epidermal cells of the embryo (Labouesse et al., 1994). The transgenic lines *plin-26::LifeAct::GFP* and *plin-26::LifeAct::mCherry* (Havrylenko et al., 2015) were crossed into different mutants, depending on the chromosomes where the genes are found. We detected increased F-actin in *wsp-1*(*gm324*) and *rga-8*(*pj60*) mutants in leading-edge cells undergoing ventral enclosure, relative to controls (Fig. 4A,B,E,G). Similarly, depleting *cdc-42* via RNAi, and measuring F-actin only in embryos that escape early arrest and differentiate epidermis, showed elevated epidermal F-actin levels (Fig. 4A,B). *toca-2; toca-1* double mutants did not significantly alter epidermal F-actin levels (Fig. 4G). Therefore, a proposed CDC-42 pathway that includes RGA-8 and WSP-1 appears to be required to maintain appropriate F-actin levels in the migrating epidermal cells.

#### Protrusions dynamics are not significantly affected by the CDC-42 pathway

Ouellette and colleagues showed decreased membrane displacement by the leading cells in *wsp-1* mutants, (Ouellette et al., 2016), although they did not examine F-actin levels or dynamics. We examined the rate of formation of protrusions and retractions, at the leading edge of leading cells during enclosure. We detected no significant changes in the rates, though the number of protrusions was slightly increased in *wsp-1*(*gm324*) and *rga-8*(*pj60*) mutants (Fig. 4C,D,H,I). Therefore, the elevated F-actin levels seem to not significantly perturb actin dynamics.

#### Speed of ventral enclosure is affected by the CDC-42 pathway

To test how changes in F-actin were affecting morphogenesis, we used the speed of migration as a phenotypic readout. To monitor timing for ventral enclosure using the epidermal F-actin movies, we measured from the time of the first protrusion to the first meeting at the ventral midline for the contralateral leading cells. *rga-8*(*pj60*) epidermal leading-edge cells migrate at slower rate (48 min) compared to the wild type (24 min), while *wsp-1*(*gm324*) (35 min) showed delays relative to controls that were statistically significant (Fig. 4J).

### RGA-8 and CDC-42 pathway regulate NMY-2/myosin in epidermal cells

**Fig. 5.**
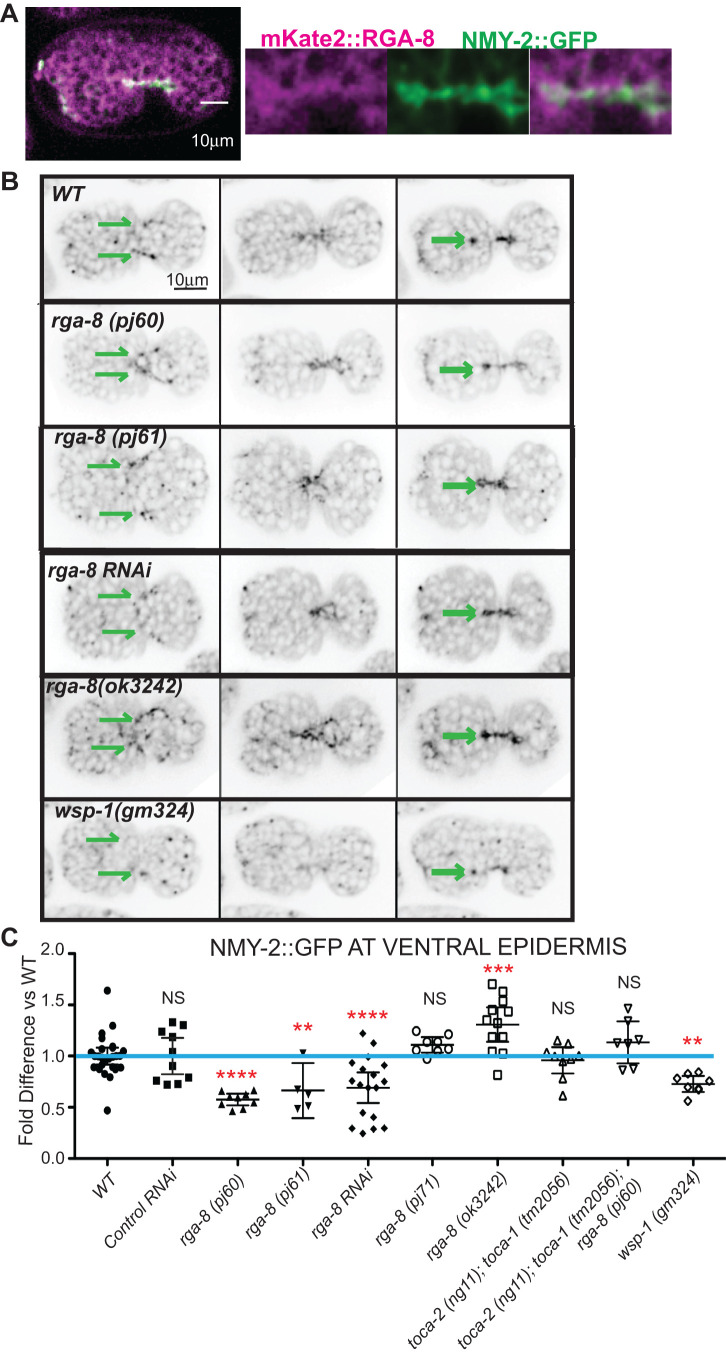
**RGA-8 and WSP-1 regulate myosin in migrating epidermal cells.** (A) 300 min embryo expressing both NMY-2/myosin II CRISPR allele, *nmy-2::gfp* (Dickinson et al., 2013), and *mKate2::rga-8*(*pj66*)*,* shown ventral up, and enlargement of the ventral midline during epidermal ventral enclosure. (B) Control and mutant strains are shown at 280, 290 and 300 min, as the epidermal cells move toward and meet at the ventral midline. The signal from *plin-26::LifeAct::mCherry* was used to verify the cells shown are in the same plane as the epidermal cells. Parallel green arrows indicate the leading edge cells of the migrating epidermis. (C) *nmy-2::gfp* expression was measured when the contralateral pocket cells meet (approximately 300 min in controls). More details are in the Materials and Methods. Results are plotted as fold difference relative to controls. Significance was calculated using one-way ANOVA with Dunnett post-test.

While CDC-42 has been connected to ventral enclosure, it is not clear which effectors of CDC-42 are responsible for this. CDC-42 regulates polarity (along with PAR-3/PAR-6/PKC) (Reviewed in Goldstein and Macara, 2007), cellular trafficking (through dynamin) (Reviewed in Grant and Donaldson, 2009), actin nucleation (through WSP-1 and formins) (Lechler et al., 2001) and myosin contractility (through the myosin kinase MRCK-1) (Gally et al., 2009). Since changes in the *cdc-42* pathway altered F-actin levels and slightly affected dynamics, we examined if RGA-8 is involved in NMY-2/myosin II regulation during ventral enclosure. Crossing *mKate2::rga-8* with the *nmy-2::gfp* CRISPR allele (Dickinson et al., 2013) showed both are expressed in the ventral epidermis during enclosure (Fig. 5A).

Myosin is enriched as puncta at the front of migrating epidermal pocket cells that later converge at the ventral midline as the contralateral pocket cells meet (Wernike et al., 2016; Wallace et al., 2018) (Fig. 5A inset; green arrows in Fig. 5B). NMY-2/myosin II enrichment is highest in the ventral epidermis when the pocket cells meet, and then drops to basal levels as the embryo further elongates. To test for *cdc-42* pathway effects on this highly regulated myosin population, we measured levels of NMY-2/myosin II at first meeting of the contralateral pocket cells, as previously reported (Wallace et al., 2018). *wsp-1*(*gm324*) pocket cells consistently showed a 20% reduction compared to wild type (Fig. 5B,C). Similarly, two *rga-8* putative null mutants (*pj60, pj61*) caused a 40% reduction compared to wild type. Interestingly, removing both TOCA-1 and TOCA-2 did not significantly change NMY-2/myosin II enrichment, while a triple mutant that removes all three, *toca-2*(*ng11*)*; toca-1*(*tm2016*) *rga-8*(*pj60),* restored the level of NMY-2/myosin II back to wild-type levels (Fig. 5C). The small deletion of *rga-8, ok3242,* predicted to truncate the C terminus starting at the GAP domain, caused a 30% increase in myosin II/NMY-2, while the GAP point mutation, *rga-8*(*pj71*) caused a small increase that was not statistically significant (Fig. 5B,C). Thus, RGA-8 and CDC-42 pathway proteins regulate accumulation of myosin II/NMY-2 in enclosing epidermal cells.

## DISCUSSION

The GTPase activating protein (GAP) family consists of many members to regulate a smaller number of RHO-GTPases. In *C. elegans*, there are only seven RHO-GTPases in comparison to 23 GAPs (Neukomm et al., 2011). Numerous studies have contributed to our understanding of how different GAPs work on the RHO-family GTPases in *C. elegans* for early embryonic polarity establishment and cytokinesis (Kumfer et al., 2010; Beatty et al., 2013), clearance of corpses during cell death (Neukomm et al., 2011), as well as epidermal morphogenesis (Ouellette et al., 2016; Zaidel-Bar et al., 2010). However, the function of the majority of the *C. elegans* GAP proteins is still unknown. This could be due to the mild phenotypes when individual GAPs are removed from the system. Some studies therefore use overexpression of these GAPs to investigate their function. Many *C. elegans* GAPs have no phenotypes on their own, so they are only examined in combination with loss of other proteins, for example, with hypomorphic alleles of the Cadherin component alpha catenin/*hmp-1* (Zaidel-Bar et al., 2010). In disease studies, GAPs are often over-expressed, rather than missing. For example, high expression of homologs of RGA-8, including SH3BP1 (also named Nadrin and ARHGAP17) is associated with more invasive and chemo-resistant cervical cancer (Wang et al., 2018), and liver cancer (Tao et al., 2016). These findings underscore the importance of analyzing GAP function using both loss and gain of function alleles.

In this study, we focused on the role of RhoGAP RGA-8, a proposed new regulator of CDC-42, during epidermal morphogenesis, and addressed two key questions: (1) how does it affect epithelial morphogenesis; and (2) which CDC-42-dependent events does this proposed GAP protein regulate? We present evidence that RGA-8 regulates active CDC-42, and that it functions together with CDC-42 to regulate ventral enclosure by regulating the level of actin and non-muscle myosin (NMY-2) in the migrating embryonic epidermal cells. These changes correlate with effects on the speed of morphogenetic events. We further showed that RGA-8 alters membrane distribution and levels of active CDC-42 and apical morphogenesis in two other epithelia, the pharynx and intestine. We propose that the RhoGAP RGA-8 regulates morphogenesis through effects on CDC-42, based on genetic interactions, expression patterns, and phenotypes.

### Genetic interactions – embryonic lethality

**Fig. 6.**
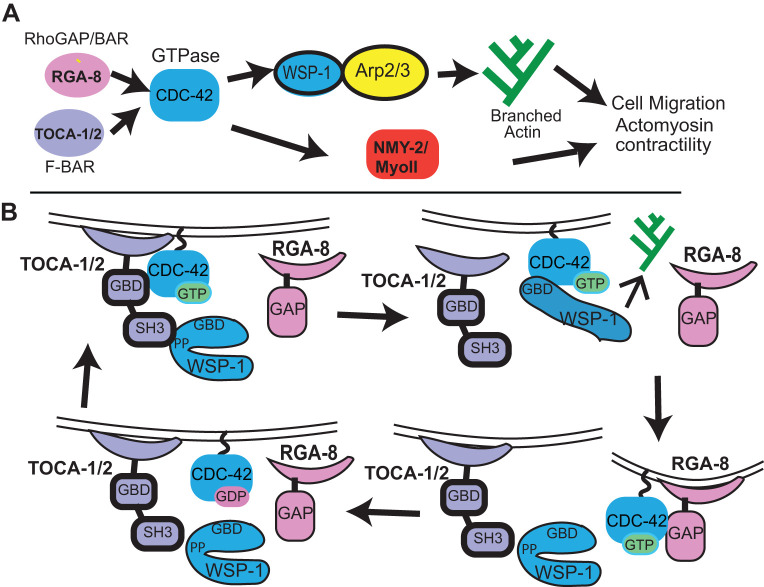
**Model for RGA-8 regulation of CDC-42.** (A) A genetic model for how the RhoGAP RGA-8 may work with TOCA-1 and TOCA-2 to regulate CDC-42 so that it can promote correct branched actin formation through WSP-1 and support myosin enrichment. (B) GAPs are proposed to fine-tune the correct enrichment of CDC-42 by localized inhibition of active CDC-42 (reviewed in Pichaud et al., 2019). A molecular model, inspired by (Watson et al., 2016), to explain how a RhoGAP with BAR domain may work sequentially to TOCA-1/ TOCA-2 F-BAR protein to support the cycle of CDC-42 activation that is needed during morphogenesis. TOCA at membranes binds active CDC-42-GTP, through its GTPase binding domain (GBD), also known as HR1 domain, and binds the Poly-Pro (PP) motif of auto-inhibited WASP/WSP-1 through its SH3 domain, promoting CDC-42-GTP association with its effector WSP-1, which also has a GBD. The BAR domain of RGA-8 may bind differently curved membranes, where it binds active CDC-42 with its GAP domain, hydrolyzes GTP to GDP, thus regenerating CDC-42-GDP, and limiting regions of active CDC-42.

*rga-8(pj60)* resulted in additive effects with the *toca-2(ng11);toca-1(tm2056)* double mutant. TOCAs are known interactors of CDC-42, that regulate branched actin by activating WSP-1/WASP. Interestingly, *wsp-1(gm324); rga-8(pj60)* double mutants resulted in intermediate embryonic lethality closer to that of *wsp-1(gm324)* mutants. In contrast, *rga-8(pj60)* enhanced lethality caused by loss of *mrck-1*, another effector of CDC-42 (Table S1). These genetic interactions suggested TOCA-1/TOCA-2 and RGA-8 mutually regulate CDC-42, likely through effects on WSP-1 (Fig. 6A, Table 1, Table S1).

### Expression patterns

A CRISPR tagged mKate2::RGA-8 strain showed that while RGA-8 is broadly and diffusely expressed, the highest expression was in regions of epithelia where GFP::CDC-42 is also enriched, including apical pharynx and intestine. This enrichment begins in embryos as these tissues polarize and mature, and continues into adulthood (Fig. 2A–D). By comparison, active CDC-42, as shown by the *Pcdc-42::gbd-wsp-1::gfp* CDC-42 biosensor, has a different, complimentary pattern in the developing intestine (Figs 2F and 3). Pharyngeal and intestinal distribution and levels of *Pcdc-42::gbd-wsp-1::gfp* depend on RGA-8 (Fig. 3), as does apical morphogenesis (Fig. 1C,E). Despite showing changes in apical/basal membrane distribution and levels of *gbd-wsp-1::gfp*, most null *rga-8* embryos are viable, suggesting the effects of RGA-8 on CDC-42 are redundant with other proteins, perhaps other GAPs. Since RGA-8 has a BAR domain, we investigated whether it localized to specific subcellular membranes. We found it has diffused apical enrichment in the pharyngeal and intestinal epithelia, and appears basal to the apical junctions in epidermal membranes (Fig. 2A–E).

### Shared phenotypes with CDC-42 pathway

The phenotypes of four *rga-8* alleles and of *cdc-42* pathway components suggested a common function during epithelial morphogenesis. In particular, we found that deletion alleles of the *toca-2; toca-1* double, or *rga-8,* or *wsp-1,* or mild (day 2) *cdc-42* RNAi, led to embryonic lethality with most arrests occurring during ventral enclosure or elongation (Fig. 1B), and defects in apical epithelia, including expanded pharyngeal and intestinal apical lumen (Fig. 1C,E). Mutations in RGA-8 led to highly penetrant changes in epidermal cell migration timing, in levels of epidermal F-actin and in levels of epidermal myosin (Figs 4 and 5). Similar changes were also seen in mutations of *toca-2;toca-1*, *wsp-1*, and *cdc-42 RNAi*. Embryonic lethality was not fully penetrant for any of the RGA-8 alleles. This is also true for complete loss of *toca-2;toca-1* (13%) or *wsp-1* (35%). One explanation for this ability of embryos to sometimes survive without the contribution of the TOCAs/RGA-8/CDC-42/WSP-1 pathway is that this pathway is responsible for only part of the events of epidermal morphogenesis. In contrast, the CED-10/WAVE pathway leads to 100% embryonic lethality, suggesting that Rac/WAVE regulation of Arp2/3 cannot be compensated for by other pathways. In addition, CDC-42 has additional proposed roles besides regulating branched actin through WSP-1, including regulation of formins, and thus linear actin, and regulation of Myosin through two kinases, PAK-1 and MRCK-1 (Gally et al., 2009). Thus, CDC-42's full contribution to epidermal morphogenesis will require analysis of additional regulatory proteins.

### Actin phenotypes

A well-studied function of CDC-42 is to regulate branched actin dynamics via the WASP/WSP-1 protein. Reduction of *cdc-42* by RNAi under the conditions used here caused some embryos to fail elongation, and some embryos to fail ventral enclosure. Similar defects were seen for the *wsp-1* deletion, *gm324*. If we assume that *wsp-1*(*gm324*) represents defective CDC-42-dependent branched actin, then the resulting elevated levels of F-actin and decreased myosin during ventral enclosure suggest branched actin properly assembled by CDC-42/WSP-1 normally interacts with and promotes myosin to support the migrations. In the one cell embryo, CDC-42 promotes actomyosin-driven cortical flows that help maintain polarity by stabilizing the anterior PARs (Gotta et al., 2001; Kumfer et al., 2010). The elevated F-actin and decreased myosin shown here in migrating epidermal cells may reflect a defect in the maintenance of polarity so that reduced myosin is unable to maintain the dorsal/ventral polarity. Alternatively, reduced myosin may be unable to remodel F-actin to promote the migrations. Overall polarity seems unaffected, since we saw ventrally polarized enrichment of F-actin and relatively unchanged protrusion dynamics (Fig. 4). An alternative explanation for the elevated F-actin is that it represents linear F-actin, since studies in yeast suggest that in the absence of branched actin nucleators, formins take over (Suarez and Kovar, 2016).

The *toca-2;toca-1* double mutant did not significantly alter F-actin or myosin levels. However, *toca-2;toca-1* mutants are defective in ventral enclosure and elongation (Giuliani et al., 2009) and in apical morphogenesis of the pharynx and intestine (Fig. 1C,E). This may indicate TOCAs play a distinct role regulating CDC-42 compared to RGA-8. For example, TOCA-1/-2 and CDC-42 are proposed to promote trafficking, and the morphogenetic effects could be linked to their trafficking roles (Fricke et al., 2009). TOCA-2/TOCA-1 and RGA-8 may also control different populations of F-actin. While TOCA-2/TOCA-1 is thought to regulate branched actin formation through WASP/WSP-1, TOCA-2/TOCA-1 can also bind two components of the WAVE/SCAR complex, WVE-1 and ABI-1 (Giuliani et al., 2009), suggesting potential crosstalk between the WAVE/SCAR pathway and TOCAs to support epidermal migrations.

### Myosin phenotypes

Our analysis of myosin behavior in epidermal cells suggested that myosin plays a role during epidermal ventral enclosure (this study and Wallace et al., 2018). Interestingly, Oulette and colleagues reported that overexpression of CDC-42 caused a decreased protrusion rate of leading-edge cell migration, resulting in delayed ventral enclosure (Ouellette et al., 2016). Similarly, we found that loss of *rga-8* resulted in delayed leading-edge cells migration during ventral enclosure. This suggests that regulated myosin activity during epidermal ventral enclosure may be under the control of CDC-42, and RGA-8.

In support of this, *rga-8* loss led to decreased *nmy-2::gfp,* while the GAP mutations, *(pj71, ok3246)* led to increased *nmy-2::gfp* (Fig. 5), suggesting misregulated CDC-42 results in misregulated levels of myosin, and perhaps localization. There is mounting evidence that myosin transiently forms epidermal ventral structures during ventral enclosure (Wernike et al., 2016; Wallace et al., 2018). Higher resolution live imaging of myosin in the epidermis during these dynamic events may help address the exact role of myosin.

### Conclusion

Analysis of some of the 23 *C. elegans* Rho GAPs has broadened our view of how the three main Rho GTPases, Rac, Rho and CDC-42, contribute to morphogenesis. Our analysis of Rho GAP HUM-7/Myo9 showed it affects Rho signaling, and is needed to attenuate Rho in the migrating epidermal cells, to prevent excessive myosin and protrusions (Wallace et al., 2018). The Nance lab showed that RhoGAP PAC-1 supports elongation by regulating CDC-42 (Zilberman et al., 2017), while the Jenna lab showed that RhoGAP RGA-7 plays a role possibly through regulation of CDC-42 (Ouellette et al., 2016). Here we add the analysis of an intriguing RhoGAP, RGA-8, with both RhoGAP and BAR domains. By extension of the proposed role for TOCAs in bringing together WSP-1 and CDC-42 to promote active CDC-42-GTP (Watson et al., 2016), we propose RGA-8 may use its GAP domain to bind to active CDC-42-GTP, and generate CDC-42-GDP and renewal of the CDC-42 cycle (Fig. 6B). It is thought that GAP hydrolysis of GTPases is the essential regulatory step for GTPase function. For CDC-42, studies in budding and fission yeast have shown that localized activation by GEFs is not enough to generate locally restricted CDC-42 activity, and GAPs are needed to prevent spread of the active form (Reviewed in Chiou et al., 2017). In fission yeast the GTPase Ras1 requires inactivation by its GAP, or it suffers loss of spatial information (Merlini et al., 2018). Models have been proposed to explain how GAP activity can promote GTPase activity, even when the GAP and GEF for a GTPase colocalize. For example, in the one-cell embryo of *C. elegans* the levels of the GTPase RHO-1/RhoA, are modulated by its GEF ECT-2 and its GAPs RGA-3 and RGA-4, which colocalize at the anterior domain (Schonegg et al., 2007). RGA-8 has a BAR domain, which suggests that like its homolog, RICH-1, it can bind and tubulate membranes (Richnau et al., 2004; reviewed in Carman and Dominguez, 2018; Carman and Rodriguez, 2018). Our results suggest the RhoGAP RGA-8 is enriched at apical regions of developing epithelia (Fig. 2), and that it regulates membrane enrichment of a CDC-42 biosensor with distinct apical/basal distribution (Fig. 3). Loss, or a GAP mutation in, RGA-8 alters apical development in these epithelia (Fig. 1C,E). These observations suggest that the RGA-8 RhoGAP plays an important role fine-tuning the localization of active CDC-42 as organs and tissues polarize.

## MATERIALS AND METHODS

### Strains

All strains used in this study are listed in Table 2. Strains were either received from the CGC (Caenorhabditis Genetics Center, USA), the NBRP (National Bio-Resource Project, Japan), or individual labs listed below, or were generated for this study. All strains were grown at 23°C unless otherwise stated.

**Table 2.**
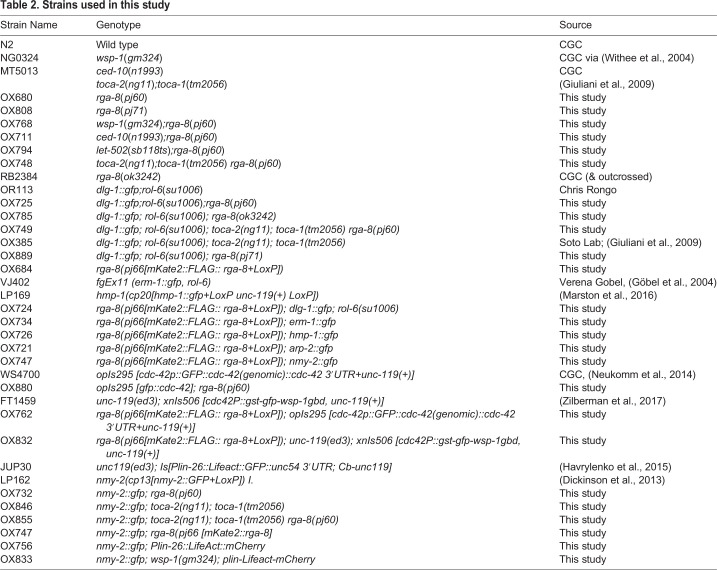
**Strains used in this study**

### Strain generation using CRISPR

CRISPR strains were made using the SEC strategy (Dickinson et al., 2015). PAM sites were identified with the help of http://crispr.mit.edu/ website from MIT as a guide. Generation of guide RNA (gRNA) plasmids was made using either sewing PCR method with the primers MSo1204-1205, MSo1304-1305 and MSo1367-1340 (Kim et al., 2014), or digestion and ligation using the primers MSo1585-1586 (Arribere et al., 2014). To test Cas9 cutting and the gRNAs we used co-CRISPR (Kim et al., 2014). To generate tagged genes, we used the plasmid rescue method from the Goldstein lab (Dickinson et al., 2015). Briefly, the 5′ flanking arms were generated using primers MSo1402-1403 and the 3′ flanking arms were generated using primers MSo1404-1405. Both arms were cloned into the pDD285 vector using Gibson assembly kit from NEB Catalogue number E2611. To generate deletion mutants, we used co-CRISPR method by the Fire lab to select for worms that have a successful Cas9 cutting event (Arribere et al., 2014). To generate specific point mutants, verified sgRNA and rescue oligos containing the desired point mutation as well as mutated PAM site were co-injected with *dpy-10* sgRNA. Silent mutation that inserted a new AvaI restriction enzyme site was engineered on the rescue oligos (MSo1602) to ease the screening process of identifying the mutants. DNA sequencing was done to verify the mutations and inserts for all strains. All strains were outcrossed at least three times to minimize possible off-target effects by the gRNAs and Cas9. Primers used for strain generation are listed in Table 3.

**Table 3.**
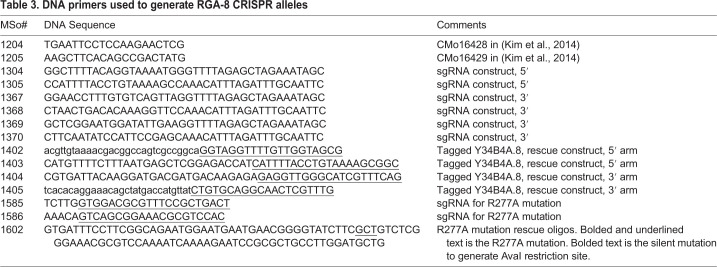
**DNA primers used to generate RGA-8 CRISPR alleles**

### RNAi experiments

All RNAi bacterial strains used in this study were administered by the feeding protocol as in Bernadskaya et al. (2012). RNAi clones were obtained from the Driscoll lab copy of the Ahringer library or generated in the lab. RNAi feeding experiments were done at 23°C unless otherwise mentioned. Worms were synchronized and transferred onto seeded plates containing RNAi-expressing bacteria. Embryos were scored for lethality at either 48 h (for *gex-3* and *cdc-42* RNAi) or 72 h (for all other genes).

### Live imaging

For all live imaging shown, embryos at the two-to-four-cell stage were dissected from adult hermaphrodites and mounted onto 3% agarose pads, covered with number 1.5 cover slip, and sealed with Vaseline. Embryos were then incubated at 23°C for 240 min. Imaging was done in a temperature-controlled room set to 23°C on a Laser Spinning Disk Confocal Microscope with a Yokogawa scan head, on a Zeiss AxioImager Z1m Microscope using the Plan-Apo 63X/1.4NA or Plan-Apo 40X/1.3NA oil lenses. Images were captured on a Photometrics Evolve 512 EMCCD Camera using MetaMorph software, and analyzed using ImageJ. Some images (Fig. 1) were done on a Zeiss Axioskop 2 microscope, using Plan-Apo 40X/1.3NA oil lens, and captured with a Roper camera. Controls and mutants were imaged within 3 days of each other with the same imaging conditions. All measurements were performed on raw data. For fluorescent measurements, background intensity was subtracted by using a box or line of the same size and measuring average intensity in the same focal plane, near the embryo. Actin intensity measurements, protrusion and retraction analysis was performed as described previously (Wallace et al., 2018) on embryos imaged at 2-min intervals for at least 120 min beginning at 240 min after the two-to-four-cell stage.

### Myosin measurements

To compare myosin levels as pocket cells meet, a line was drawn through the enclosing the pocket cells as they first touch (approximately 300 min) (green arrows in Fig. 5B). Time points shown and measured are those when the epidermal cells first touch based on epidermal F-actin signal. To measure myosin puncta on the same focal plane as the epidermis, we co-localized the highest Plin-26::LIFEACT::mCherry intensity with NMY-2::GFP and recorded the highest of three NMY-2::GFP measurements per embryo (Fig. 5B,C). Maximum intensity values were recorded after subtracting the average background fluorescence. The graph in Fig. 5C records the relative level of NMY-2::GFP, after normalization of either WT, or WT on control RNAi, to 1.

### Statistical analysis

Error bars show 95% confidence intervals. Asterisks (*) denote *P* values *P<0.05, **P<0.001, ***P<0.0001, ****P<0.00001. All statistical analysis was performed using GraphPad Prism. Specific tests used for each data set are included in the Figure legend.

## Supplementary Material

Supplementary information
