# Supplementary material for: RhoGAP RGA-8 supports morphogenesis in C. elegans by polarizing epithelia
Source: Biol Open. 2020 Nov 26;9(11):bio056911. doi: 10.1242/bio.056911 (PMC7710025; doi:10.1242/bio.056911)
Supplement: Supplementary information [file biolopen-9-056911-s1.pdf]

Table S1. Raw Genetics Counts with Phenotypes

	Counts								
**Genotype**	Date	Early arrest	Ven Enc	Elongation	Live	**Tot dead**	**n**	% Lethality	Stand. Deviation
N2 Control	7/2/19	1	2	4	366	7	373	0.02	
	7/3/19	2	9	9	688	20	702	0.03	
	7/1/19	0	10	2	392	12	404	0.03	
	**TOT**	**3**	**21**	**15**	**1446**	**39**	**1479**	0.026	0.0058
rga-8(pj60)	17-Jul	1	1	1	86	3	89	0.034	
	18-Jul	1	1	1	86	3	89	0.034	
	29-Aug	0	2	1	161	3	164	0.018	
	26-Sep	1	5	4	209	10	219	0.046	
	TOT	3	9	7	542	19	561	0.034	0.0112
rga-8(pj71)	18-Jul	2	9	8	131	19	150	0.127	
	21-Aug	1	8	10	203	19	222	0.086	
	27-Aug	0	7	4	121	11	132	0.083	
	12-Sep	1	38	14	227	53	280	0.189	
	TOT	4	62	36	682	102	784	0.130	0.0496
rga-8(pj61)	10-Feb	0	5	3	121	8	129	0.062	
	12-Feb	1	4	0	40	5	45	0.111	
	2/6/20	0	2	13	439	15	454	0.033	
	TOT	1	11	16	600	28	628	0.045	0.0395
wsp-1(gm324)	21-Aug	2	64	2	176	68	244	0.279	
	27-Aug	16	119	20	250	155	405	0.383	
	12-Sep	9	79	16	195	104	299	0.348	
	TOT	27	262	38	621	327	948	0.345	0.0529
toca-2(ng11);toca-1(tm2056)	21-Aug	1	4	12	164	17	181	0.094	
	27-Aug	1	4	6	104	11	115	0.096	
	30-Oct	1	31	51	439	83	522	0.159	
	TOT	3	39	69	707	111	818	0.136	0.0371

Biology Open • Supplementary information

rga-8(pj60); wsp-1	5-Sep	0	4	5	61	9	70	0.129	
	12-Sep	1	42	39	313	82	395	0.208	
	13-Sep	1	7	14	117	22	139	0.158	
	17-Sep	1	27	34	156	62	218	0.284	
	TOT	3	80	92	647	175	822	0.213	0.0681
rga-8(pj60); toca-2; toca-1	5-Sep	1	18	4	123	23	146	0.158	
	12-Sep	0	16	8	70	24	94	0.255	
	13-Sep	0	6	7	92	13	105	0.124	
	20-Sep	1	12	5	56	18	74	0.243	
	TOT	2	52	24	341	78	419	0.186	0.0644
dlg-1::gfp - Control	5/6/19	2	2	6	607	8	615	0.013	
	5/8/19	0	0	0	60	0	60	0.000	
	TOT				667	8	675	0.012	0.0092
rga-8(pj71); dlg-1-gfp	27-Aug	1	4	7	229	12	241	0.050	
	12-Sep		39	21	377	60	437	0.137	
	TOT	1	43	28	606	72	678	0.106	0.0619
rga-8(ok3246); dlg-1-gfp	18-Jul	4	5	11	197	20	217	0.092	
	21-Aug	0	15	17	225	32	287	0.111	
	28-Aug	0	1	11	242	12	254	0.047	
	TOT	4	21	39	664	64	758	0.084	0.0330
wsp-1; dlg-gfp	27-Aug	11	65	24	127	100	227	0.441	
	12-Sep	6	35	12	155	53	208	0.255	
	18-Sep	5	129	46	520	180	700	0.257	
	TOT	22	229	82	802	333	1135	0.293	0.1066
toca-2; toca-1; dlg-1::gfp	27-Aug	1	29	27	48	57	105	0.543	
	12-Sep	0	9	10	48	19	67	0.284	
	20-Sep	3	40	38	185	81	266	0.305	

	TOT	4	78	75	281	157	438	0.358	0.1440
toca-2;pj60;dlg-gfp	27-Aug	4	17	16	220	37	257	0.144	
	29-Aug	3	42	13	148	58	206	0.282	
	12-Sep	1	10	4	90	15	105	0.143	
	13-Sep	4	15	16	143	35	178	0.197	
	17-Sep	2	12	10	134	24	158	0.152	
	TOT	14	96	59	735	169	904	0.187	0.0592
rga-8 RNAi									
	21-Oct	1	2	4	288	7	295	0.024	
	24-Oct	0	3	4	182	7	189	0.037	
	24-Oct	0	2	24	265	26	291	0.089	
TOT		1	7	32	735	40	775	0.052	0.0347
rga-8 RNAi Day3; toca-2-1	23-Sep	2	37	8	80	47	127	0.370	
	26-Sep	2	29	13	102	44	146	0.301	
	21-Oct	0	3	0	23	3	26	0.115	
	24-Oct	0	25	28	170	53	223	0.238	
	24-Oct	1	9	5	71	15	86	0.174	
TOT		9	103	54	446	253	881	0.287	0.1007
rga-8 RNAi; wsp-1	26-Sep	3	21	6	60	30	90	0.333	
	21-Oct	0	16	1	24	17	41	0.415	
	24-Oct	4	30	15	138	49	187	0.262	
	24-Oct	5	57	13	164	75	239	0.314	
TOT		12	124	35	386	171	557	0.307	0.0634
rga-8 RNAi/N2 Day 3	3-Mar	0	2	14	406	16	422	0.038	
rga-8 RNAi/N2 Day 3	9-Mar	4	1	6	234	11	245	0.045	
rga-8 RNAi/N2 Day 3	18-Mar	0	0	7	185	7	192	0.036	
rga-8 RNAi Day 3	26-Mar	0	2	6	179	8	187	0.043	
rga-8 RNAi/N2 Day 3	3/6/20	0	7	9	271	16	287	0.056	
	TOT	4	12	42	1275	58	1333	0.044	0.0076

rga-8 RNAi/wsp-1		4	32	22	163	58	221	0.262	
rga-8 RNAi/wsp-1		3	53	26	172	82	254	0.323	
rga-8 RNAi/wsp-1		5	45	23	145	73	218	0.335	
rga-8 RNAi wsp-1		1	14	11	30	26	56	0.464	
rga-8 RNAi wsp-1		1	10	10	71	21	92	0.228	
	TOT	14	154	92	581	260	841	0.309	0.0905
rga-8 RNAi/toca-2/1		1	49	31	94	81	175	0.463	
rga-8 RNAi/toca-2/1		0	4	7	20	11	32	0.344	
rga-8 RNAi/toca-2/1		1	4	5	23	10	33	0.303	
rga-8 RNAi/toca-2/1		1	15	26	88	42	130	0.323	
	TOT	3	72	69	225	144	370	0.389	0.0717
rga-8 RNAi/rga-8(pj60)		0	9	12	353	21	374	0.056	
	TOT	0	9	12	353	21	374	0.056	
RNAi/ Genotype	Date	Early arrest	Ven Enc	Elongation	Live	Tot dead	n		
cdc-42 RNAi /N2 Day 2	12-Feb	0	26	13	42	49	91	0.538	
	14-Feb	0	126	68	175	194	369	0.526	
	10-Mar	1	59	18	44	78	122	0.639	
	17-Mar	0	16	27	36	43	79	0.544	
	TOT	1	227	126	297	364	661	0.551	0.0522
cdc-42 RNAi/pj60	12-Feb	1	24	31	64	56	120	0.467	
	14-Feb	0	34	37	60	71	131	0.542	
	10-Mar	0	75	30	49	105	154	0.682	
	17-Mar	0	14	15	30	29	59	0.492	
	TOT	1	147	113	203	261	464	0.563	0.0961
cdc-42 RNAi/pj61	12-Feb	0	17	14	14	31	45	0.689	
	14-Feb	0	55	26	38	81	119	0.681	
	10-Mar	0	306	21	40	327	367	0.891	

	17-Mar	0	60	192	126	252	378	0.667	
	TOT	0	438	253	218	691	909	0.760	0.1065
cdc-42 RNAi/pj71	12-Feb	0	58	34	32	92	124	0.742	
	14-Feb	0	55	34	43	89	132	0.674	
	10-Mar	2	230	38	48	270	318	0.849	
	17-Mar	0	160	46	45	206	251	0.821	
	TOT	2	503	152	168	657	825	0.796	0.0791
cdc-42 RNAi/toca-1/2	12-Feb	0	21	8	1	29	30	0.967	
	14-Feb	4	19	13	29	36	65	0.554	
	10-Mar	0	66	35	44	101	145	0.697	
	TOT	4	106	56	74	166	240	0.692	0.2097
cdc42 RNAi/wsp-1	10-Mar	0	84	40	70	124	194	0.639	
	17-Mar	2	117	31	33	150	183	0.820	
	TOT	2	201	71	103	274	377	0.727	0.7294
gex-3 RNAi Day 2	25-Mar	0	3	4	13	7	20	0.350	
gex-3 RNAi Day 2	26-Mar	0	30	26	50	56	106	0.528	
	TOT	0	33	30	63	63	126	0.500	0.1261
gex-3/pj60	25-Mar	0	132	108	143	240	383	0.627	
gex-3/pj60	26-Mar	0	214	28	25	242	267	0.906	
	TOT	0	346	136	168	482	650	0.742	0.1978
gex-3/pj61	25-Mar	0	49	40	41	89	130	0.685	
gex-3/pj61	26-Mar	2	221	57	19	280	299	0.936	
	TOT	2	270	97	60	369	429	0.860	0.1781
gex-3/pj71	25-Mar	0	12	18	13	30	43	0.698	
gex-3/pj71	26-Mar	0	208	13	6	215	221	0.973	
	TOT	0	220	31	19	245	264	0.928	0.1946

Biology Open • Supplementary information

mrck-1 RNAi	23-Sep	0	2	2	37	4	41	0.098	
	26-Sep	0	16	27	152	43	195	0.221	
	TOT	0	18	29	189	47	236	0.199	0.0869
mrck-1 RNAI; pj60	23-Sep	1	36	60	257	97	354	0.274	
	26-Sep	1	35	43	222	79	301	0.262	
	TOT	2	71	103	479	176	655	0.268	0.0082
ced-10(n3246)					598	177	775	0.230	
	TOT					177	775	0.230	
ced-10(n3246); pj60	11-Feb	0	27	20	95	47	142	0.331	
		1	25	32	126	58	184	0.315	
	10-Feb	5	61	17	118	83	201	0.413	
	TOT	6	113	69	339	188	527	0.357	0.0525
ced-10(n1993)					633	75	708	0.106	
	TOT					75	708	0.106	
ced-10(n1993); pj60	11-Feb	0	50	33	389	83	472	0.176	
	16-Feb	3	1	2	89	6	95	0.063	
	10-Feb	0	5	10	61	15	76	0.197	
	TOT	3	56	45	539	104	643	0.162	0.0721
N2 let-502 RNAi Day 3	5/25/19	1	19	27	294	47	241	0.195	
	5/2/19	8	28	3	163	39	202	0.193	
	4/29/19				270	333	603	0.552	
	TOT	9	47	30	727	419	1046	0.401	0.2068
pj60 let-502 RNAi Day 3	5/25/19	2	22	44	78	68	146	0.466	
	5/2/19	27	146	107	102	280	382	0.733	
	4/29/19				90	126	216	0.583	
	TOT	29	168	151	270	474	744	0.637	0.1339

p71 let-502 RNAi Day 3	5/2/19	8	77	36	146	121	267	0.453	
	4/29/19				160	211	371	0.569	
	TOT	8	77	36	306	332	638	0.520	0.0817
mKate2::rga-8(pj66) #2109	5/15/19	1	0	1	207	2	209		
mKate2::rga-8(pj66) #2108	5/15/19	0	0	1	175	1	176		
	TOT					3	385	0.01	
mrck-1 RNAi	23-Sep	0	2	2	37	4	41	10%	
	26-Sep	0	16	27	152	43	195	22%	
	TOT	0	18	29	189	47	236	20%	0.084852814
mrck-1 RNAI; rga-8(pj60)	23-Sep	1	36	60	257	97	354	27%	
	26-Sep	1	35	43	222	79	301	26%	
	TOT	2	71	103	479	176	655	27%	0.007071068
